# Three distinct pneumotypes characterize the microbiome of the lung in BALB/cJ mice

**DOI:** 10.1371/journal.pone.0180561

**Published:** 2017-07-06

**Authors:** Julia Scheiermann, Dennis M. Klinman

**Affiliations:** Cancer and Inflammation Program, National Cancer Institute, Frederick, Maryland, United States of America; University of Illinois at Urbana-Champaign, UNITED STATES

## Abstract

Bacteria can rarely be isolated from normal healthy lungs using conventional culture techniques, supporting the traditional belief that the lungs are sterile. Yet recent studies using next generation sequencing report that bacterial DNA commonly found in the upper respiratory tract (URT) is present at lower levels in the lungs. Interpretation of that finding is complicated by the technical limitations and potential for contamination introduced when dealing with low biomass samples. The current work sought to overcome those limitations to clarify the number, type and source of bacteria present in the lungs of normal mice. Results showed that the oral microbiome is diverse and highly conserved whereas murine lung samples fall into three distinct patterns. 33% of the samples were sterile, as they lacked culturable bacteria and their bacterial DNA content did not differ from background. 9% of samples contained comparatively higher amounts of bacterial DNA whose composition mimicked that detected in the URT. A final group (58%) contained smaller amounts of microbial DNA whose composition was correlating to that of rodent chow and cage bedding, likely acquired by inspiration of food and bedding fragments. By analyzing each sample independently rather than working with group averages, this work eliminated the bias introduced by aspiration-contaminated samples to establish that three distinct microbiome pneumotypes are present in normal murine lungs.

## Introduction

Next generation sequencing (NGS) studies suggest that bacteria can be found throughout the human body and that the composition of this microbiome influences physiologic and pathologic processes ranging from immunity to tissue repair to disease susceptibility [1–4]. Historically, the lungs were considered to be sterile as bacteria could rarely be cultured from normal pulmonary tissue. This expectation of sterility resulted in the lungs being excluded from the original Human Microbiome Project. Yet recent NGS studies suggest that mammalian lungs contain bacterial DNA from phyla including *Proteobacteria*, *Firmicutes*, *Actinobactera*, *Bacterioidetes* and *Cyanobacteria* with the most abundant genera being *Fusobacteria*, *Staphylococcus*, *Streptococcus*, *Lactobacillus*, *Rastonia*, *Enterobacteria*, *Sphingomonas*, *Pasteurella*, *Massalia*, *Corynebacteria* and *Pseudomonas* (S1 Table) [5–11].

The etiology and progression of respiratory illnesses including chronic obstructive pulmonary disease and cystic fibrosis may be influenced by the pulmonary microbiome [12;13]. Efforts to clarify the relationship between bacterial colonization of the lungs and disease rely on murine models, underscoring the importance of defining the pulmonary microbiome in mice (see [14] for a detailed discussion). While several groups report that microbes from the oral cavity are the dominant source of bacteria in the lungs [15;16], no study comparing the microbiota of the lungs vs oral cavity in mice has been published. Moreover, potential artifacts introduced by DNA contamination of samples and reagents complicate interpretation of available data, leaving this a topic of ongoing interest and debate [17].

The current work was undertaken to characterize the number, type and source of bacteria present in the lungs of Balb/cJ mice. Results suggest that there is no "typical" lung microbiome. Rather, three distinct pneumotypes were detected. A third of the tissue samples were sterile, consistent with the failure of traditional culturing techniques to detect bacteria in healthy lungs [6–11]. The second pneumotype contained flora characteristic of the oral cavity. The third pneumotype was composed of bacterial DNA also found in rodent chow and cage bedding. These findings suggest that the bacterial DNA content of the murine lung is more complex than previously appreciated.

## Materials and methods

### Animal housing

Female BALB/cJ mice (Charles River, Frederick, MD) were housed under SPF conditions. All studies were approved by the Institutional Animal Care and Use Committee of the NCI (protocol #14–029) and followed the National Institutes of Health guidelines for the use and care of live mice (Bethesda, MD). The mice were supplied with reverse osmosis purified water and food *ad libidum*, and maintained with light cycles of 6 am—6 pm, temperatures between 68–72°F and a humidity of between 30–50%. All mice were housed in a single large cage for at least 6 weeks prior to initiation of the study to minimize any effect of environmental factors on the microbiome.

### Tissue collection

Tissue samples from 18 animals were collected sterilely by entry through the thoracic cavity following isoflurane anesthesia of the animals and subsequent cervical dislocation. Samples were taken from the superior lobe (upper lung, UL) and inferior lobe (lower lung, LL) of the right lung (n = 36). The posterior oropharynx (URT) of the same animal was then collected surgically (n = 18). Individually sterilized and UV treated sets of surgical instruments were used for each anatomical site in each animal. The surgical instruments were autoclaved and UV treated to minimize DNA contamination. Control samples from all reagents (n = 8) were included to identify potential sources of contamination in each processing step. Additionally, “No Template Control” (NTC) water samples (n = 3) were processed in the identical manner as tissue samples. A MOCK sample with known composition was processed as a positive control.

### 16S Sequence Acquisition

Total DNA was immediately extracted from the tissue samples after collection (Pathogen Mini Kit, Quiagen, Denmark) and 16SrDNA genes sequenced using the MiSeq Sequencer (Illumina, Inc, San Diego, CA, USA) with fusion primers specific for the amplification of the entire 254 base pair V4 hyper-variable region. To compensate for the low concentration of bacterial DNA in lung samples the number PCR cycles was increased to 35. This yielded a clear 16SrDNA band in gel electrophoresis that was absent from the NTC controls. The NTCs and reagent controls were processed and sequenced in parallel with the samples. Sequence data was further processed using standard microbiome pipelines implemented in the software package Qiime [18;19] as detailed below.

### Contig building and sequence filtering

Paired sequencing reads in FASTQ format were joined by overlap (minimum of 6 bp) to form single contigs using the Qiime [18] script *join_paired_ends*.*py* and the *ea-utils* (https://github.com/ExpressionAnalysis/ea-utils). The contigs in FASTA format were assigned unique sample identifiers using the Qiime script *split_libraries_fastq*.*py*. Our analytic approach included the identification of contaminating sequences derived from reagents and water NTCs used in the amplification process and removal of such sequences using *in silico* techniques. The FASTA contigs assigned to reagent controls and NTCs were split from the sample contigs after which the sample contigs were compared to those of the reagent controls using the *usearch* program [20]. Sample contigs that matched any contig from a reagent control at the level of 99% identity over at least 250 bp were removed. Our goal was to remove only sequences that represented contaminants from the data set. Removal of background sequences at this stage rather than at the Operational Taxonomic Unit (OTU) level facilitated analysis of low biomass samples as it avoided the removal of sequences that would otherwise be discarded together with contaminants when entire OTUs are removed. The remaining sample contigs were filtered for chimeric sequences using the *uchime_ref* [20;21] with comparison against the GOLD database of chimera-free 16S sequences [22].

### OTU clustering and taxonomic assignment

Bioinformatics analyses, such as OTU clustering, were performed on sequences from URT and lung samples as a combined data set. The remaining lung and URT-derived contigs after the filtering steps were assigned to OTUs using the Qiime script *pick_open_reference_otus*.*py* to the corresponding Greengenes database [23]. Contigs matching sequences at an identity threshold of > 97% were assigned OTUs whose taxonomy was based on Greengenes. Representative sequences from the *de novo* OTUs were compared to the 16SrDNA NCBI database using BLASTn [24;25] and taxonomy determined based on alignment over the full sequence length. Bacterial OTUs were classified at the genus level. Sequences from a total of 478 bacterial entities were identified in the data set with 31 genera containing sequences at >1% in relative abundance.

### Statistical analyses

Bioinformatic analysis of the data was performed using the *R* statistical program (http://www.R-project.org) extended with several packages including *metagenomeSeq*, *survival* and *vegan* (https://CRAN.R-project.org). Alpha diversity was measured using the *Inverse Simpson* and *Chao 1* indices. To determine the beta diversity, a multivariate ANOVA procedure using permutation-based analysis of variance (ADONIS with 999 permutations) was performed on read counts of the bacterial communities from the sampling sites. *GraphPad Prism* (GraphPad Software, LA Jolla, CA), *R* and *Microsoft Excel* were used as graphical tools and for making comparisons using ANOVA and performing the Wilcox test to detect differences between groups. ANOVA was also used to calculate the contribution of sampling site and animal variability to variance as measured by the Bray-Curtis distance measurement (https://github.com/vegandevs/vegan/). Count data for taxa were normalized to proportions that totaled 100% per sample for statistical comparison. Normalized taxon abundances were compared between two sites using the Wilcox test.

"Representative" microbiomes were generated by averaging log_2_ transformed data from all members of each group. Correlation coefficients were calculated for each sample/group in comparison to this "representative" microbiome. The "overlap" between samples in a group (or between groups) was calculated as follows: the average number of sequences/genus was multiplied by the fraction of samples in which that genus was found. Data from all genera was summed, divided by the total number of sequences in all samples and multiplied by 100%.

To provide additional evidence for the proposed model of pneumotypes, unsupervised methods for grouping the microbial community were used. These included the weighted UniFrag metric using Non-Metric Miltidimensional scaling (NMDS) and the classification of samples using the Dirichlet Multinomial Mixtures modeling technique [26].

### Nucleotide sequence accession number

The raw reads used in this study are publicly available through the NCBI Sequence Read Archive (SRA) under the BioProject accession number SRP082977. Two reagent control samples and two samples from drinking water had no sequence reads and these empty data files were omitted from the BioProject.

## Results

### Experimental strategy

Experimental animals showed no physical or behavioral changes during the course of the study. Samples were surgically collected from the superior and inferior lobes of the right lung via the thoracic cavity. The microbial composition of both lung sites (UL and LL) was similar, clustering together within 95% confidence limits (S1 Fig). Such samples were therefore treated as arising from a single site denoted "lung" in subsequent analysis. Tissue was also obtained from the URT of each animal as well as from their cage bedding, drinking water and rodent chow. Individually sterilized and UV treated instruments were used for each tissue collection.

Previous studies suggest that the reagents used in microbiome analyses can contain low levels of bacterial DNA [27, 28]. Such contamination presents a major problem when evaluating low biomass/sterile samples since amplification of reagent-derived contaminants will disproportionately effect the observed outcome. To identify and remove sequences arising from such contamination, 8 reagent controls and 3 water/diluent samples were amplified and sequenced in parallel with tissue from the lung and URT. The reagent-derived samples yielded 9053 sequences that clustered into 173 OTUs. These were largely of the phyla *Proteobacteria* (80%) and *Firmicutes* (11%) and the genera *Pseudomonas* (53%) and *Escherichia* (15%), all of which were previously described as reagent contaminants (S2 Table) [27]. Sequences amplified from lung samples that were > 99% identical over a span of at least 250 bp to those present in the reagent controls were removed from the database. This resulted in the removal of 44% of the sequence reads (5.7 x 10^5^ out of 1.3 x 10^6^ total sequence reads). This filtering step decreased the relative abundances of (but did not entirely eliminate) several genera.

Consistent with previous reports, samples from the URT contained >1,000 fold more bacterial 16SrDNA than samples from the lungs (S2 Fig) [15;29].

### Microbiome of the URT

The strategy used to generate and analyze bacterial sequences was evaluated using samples obtained from the URT. 6 URT samples were excluded because errors in DNA processing. The composition of the microbiome in URT samples from co-housed mice was highly reproducible allowing for the generation of a "representative" microbiome (Table 1). When individual URT samples were compared to this "representative" microbiome (Table 2), the correlation coefficient inclusive of all genera exceeded 0.9 (p < 0.001). A second method was used to further evaluate the degree of similarity (denoted "overlap") between samples. This comparison was based on a formula that considered both the relative frequency of each bacterial genus and the fraction of samples in which that genus was present. Results indicate that the URT microbiome of different animals overlapped by > 70% (Table 3).

**Table 1:** Frequency and distribution of genera "representative" of the lung^unique^, lung^aspirate^ and URT groups.

Genera	Percent composition		
	Lung^unique^	Lung^aspirate^	URT
**Acinetobacter**	3.01	-	-
**Actinobacillus**	1.84	11.18	14.96
**Aggregatibacter**	0.72	1.13	3.83
**Brevundimonas**	1.06	0.08	-
**Candidatus**	7.03	0.21	0.01
**Chryseobacterium**	1.42	-	-
**Desulfotomaculum**	1.44	-	-
**Eubacterium**	1.73	-	-
**Flavobacterium**	6.28	0.3	0.01
**Fluviicola**	1.32	-	-
**Geobacillus**	2.05	-	-
**Haemophilus**	0.18	2	2.86
**Lactobacillus**	0.6	0.78	1.02
**Legionella**	1.5	-	-
**Mannheimia**	0.12	3	1.89
**Methylobacterium**	1.84	-	-
**Paludibacter**	1.83	-	-
**Parvularcula**	2.58	-	-
**Povalibacter**	1.1	-	-
**Pseudomonas**	2.31	-	-
**Simiduia**	2.27	-	-
**Sphingomonas**	1.32	-	-
**Stenotrophomonas**	1.91	0.16	-
**Streptococcus**	14.49	72.21	74.8
**Variovorax**	1.53	-	-

The mean relative abundance of genera present at a frequency of >1% within any group is shown. The groups lung^unique^ and lung^aspirate^ indicating two distinct pneumotypes are shown along with the URT. The group lung^aspirate^ represents a microbiome similar to that of the URT, whereas lung^unique^ shows a more distinct and diverse microbiome.

**Table 2:** Group correlation coefficients.

Sample group	Correlation Coefficient		
	Lung^unique^	Lung^aspirate^	URT
**URT**			0.93***
**Lung** ^**aspirate**^		0.86***	0.74***
**Lung** ^**unique**^	0.41***	0.21	0.17

A count matrix inclusive of all 65 genera was generated for each sample and compared to the "representative" microbiome of the lung^unique^, lung^aspirate^ and URT groups as shown in Table 1 (see Methods section for details). The mean correlation coefficient of all members of each group with the "representative" microbiome is shown.

***; p<0.001

**Table 3:** Microbiome overlap between groups.

Sample group	Percent Overlap		
	Lung^unique^	Lung^aspirate^	URT
**URT**			74
**Lung** ^**aspirate**^		67	75
**Lung** ^**unique**^	20	54	45

The similarity between microbiomes from all samples per group was calculated as described in the Methods section. In brief, the average number of sequences/genus was multiplied by the fraction of samples in which that genus was present. The outcome for all genera was summed and divided by the total number of sequences/group.

### Microbiome of the lung

In contrast to URT samples, there was considerable heterogeneity in the frequency and distribution of bacterial sequences present in low biomass lung samples. Iterative comparisons between samples showed that they fell into three discrete categories. The number and type of bacterial sequences present in a third of lung samples (33%) had fewer than 50 sequence counts and did not differ from that of the DNA-free water controls included in each study. That group is referred to as "lung^background^", as individual samples were free from bacterial DNA other than what could be ascribed to water/reagent contamination and/or PCR/sequencing artifact.

A second group of samples (58%) contained many more bacterial sequences and generated a "representative" microbiome that was larger and taxonomically diverse (Table 1). We refer to this group as "lung^unique^". A third group, referred to as "lung^aspirate^" (9% of samples), contained more bacterial sequences than the other lung samples and harbored a "representative" microbiome similar to that of the URT (while also including few genera detected in the lung^unique^ group, Table 1). It should be noted that averaging data from all samples would yield a microbiome that resembled the URT and thus would not be representative of most lung samples due to the bias introduced by comparatively large number of sequences present in the lung^aspirate^ group.

In addition, ADONIS permutation-based analysis of variance was performed. That evaluation showed that the groups identified above were significantly different from one another (p < 0.001) whereas other possible sources of variance, such as variation between the animals from which individual samples were derived, did not achieve statistical significance.

To independently examine whether the lung microbiome should be organized into three distinct groups, an unsupervised analysis of the weighted UniFrac distances between the samples was used. An NMDS analysis based on the UniFrac distances highlights the tendency of the samples to cluster according to the pneumotypes identified (S3 Fig).

### Comparison of the lung and URT microbiomes

Two statistical approaches were taken to compare the microbiomes present in lung^unique^, lung^aspirate^ and URT sample groups (lung^background^ samples were not included as they were devoid of evaluable sequences). First, a correlation coefficient for each sample was calculated in comparison to the "representative" microbiome generated for each group (Tables 1 and 2). Samples from the URT were quite homogeneous and yielded an intra-group correlation coefficient of > 0.9 (p < 0.001). Members of the lung^aspirate^ group showed a similarly high intra-group correlation coefficient of 0.86 (p < 0.001). Consistent with the URT being the dominant source of the lung^aspirate^ microbiome, the lung^aspirate^ group showed a strong correlation with that of the URT (0.74; p <0.001). There was greater mouse-to-mouse variability among members of the lung^unique^ group, such that their intra-group correlation coefficient was 0.41 (p < 0.001). The lung^unique^ samples showed a poor correlation with URT (0.17, p > 0.05) or lung^aspirate^ samples (0.21, p > 0.05) (Table 2).

The second approach used to compare the microbiomes of different groups examined their degree of "overlap". As described in the Methods section, this calculation took into account the relative frequency and fraction of mice harboring each genus. Results show that the microbial composition of URT samples overlapped by > 74%. Similarly, lung^aspirate^ microbiomes showed intra-group overlap of 67% and overlap with URT samples of 75%. The intra-group overlap of lung^unique^ samples was only 20% while overlap between lung^unique^ vs lung^aspirate^ and URT samples was 54% and 45%, respectively (Table 3).

### Analysis of groups based on PCOA clustering

**Fig 1:**
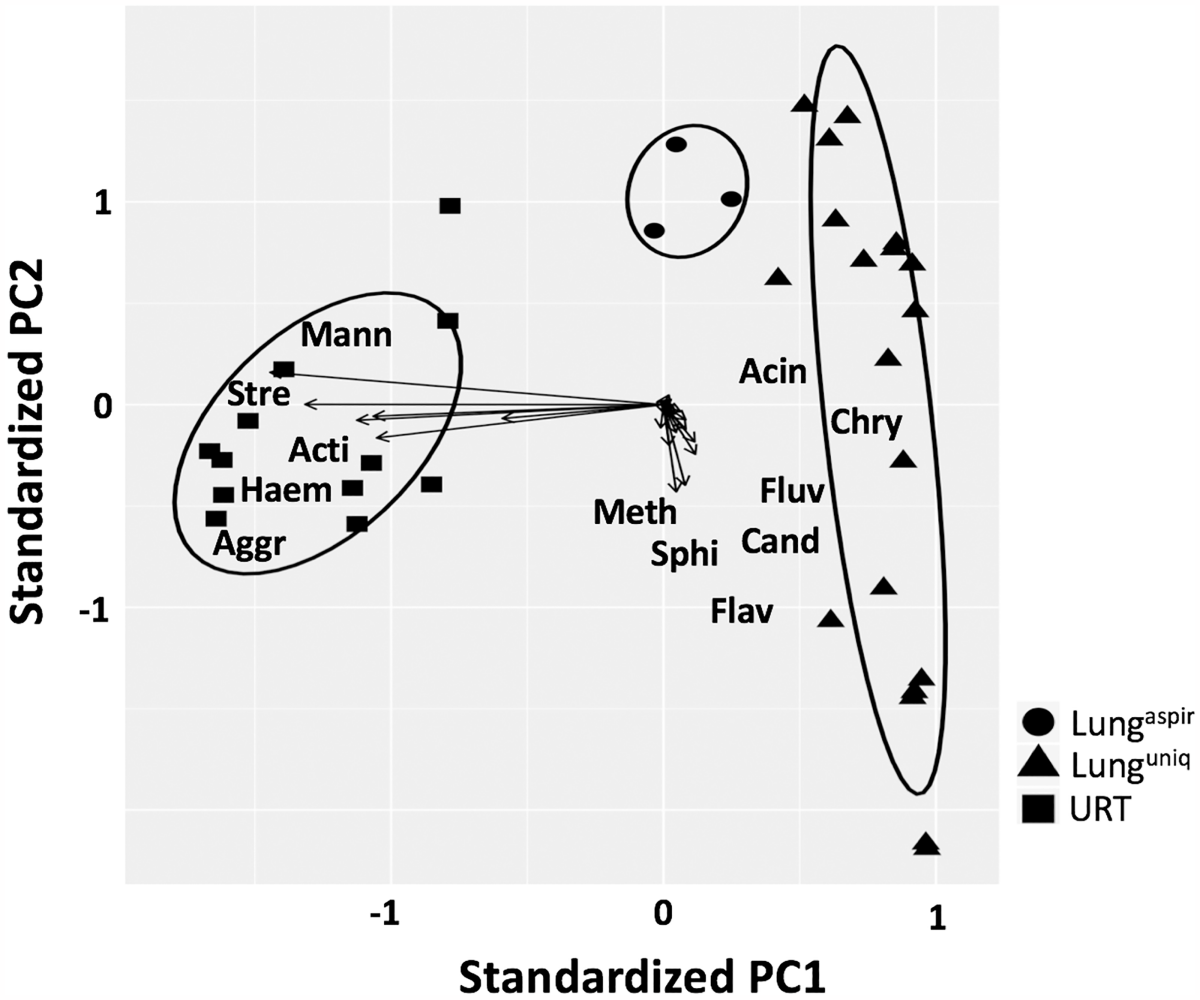
PCOA. A PCOA biplot was generated based on the log_2_ number of sequences for all key genera (77% of sample variability is captured on the X axis and 6% on the Y axis). The location of the genera relative to the sample groups is shown. 95% confidence limits of sample groups are circled. Genera abbreviations: Acin: *Acinetobacter*, Acti: *Actinobacillus*, Aggr: *Aggregatibacter*, Cand: *Candidatus*, Chry: *Chryseobacterium*, Flav: *Flavobacterium*, Fluv: *Fluviicola*, Haem: *Haemophilus*, Mann: *Mannheimia*, Meth: *Methylobacterium*, Sphi: *Sphingomonas*, Stre: *Streptococcus*.

The compositional similarities among samples were evaluated using Principle Coordinate Analysis (PCOA) (Fig 1). Consistent with the pneumotypes described above, samples from the URT (squares) clustered together and included bacteria of the genera *Streptococcus*, *Aggregatibacter*, *Actinobacillus*, *Mannheimia*, and *Haemophilus*. By comparison, lung^unique^ samples (triangles) formed an independent cluster characterized by the presence of *Acinetobacter*, *Candidatus*, *Flavobacterium*. *Fluviicola*, *Methylobacterium*, *Chryseobacterium* and *Sphingomonas*. The lung^aspirate^ group (circles) also formed a separate cluster that was located between lung^unique^ and URT along PC1.

In addition, we applied a Dirichlet Multinomial Mixture modeling analysis [26] to survey the spectrum of models constructed using from 1 to 7 components in our data set excluding the lung^background^ samples, since they had very few sequence reads to evaluate. Based on the Laplace goodness of fit parameter, where the distribution of taxa is best defined by the lowest value, optimal results were achieved using 2 components. That optimal model merged URT and lung^aspirate^ samples into a first group and identified a second group, which correspond to lung^unique^ respectively. Since the URT and lung^aspirate^ samples derived from different anatomical sites, we re-partitioned those samples into the separate groups as URT and lung^aspirate^ (S4 Fig).

### Analysis of taxonomic richness and alpha diversity by group

**Fig 2:**
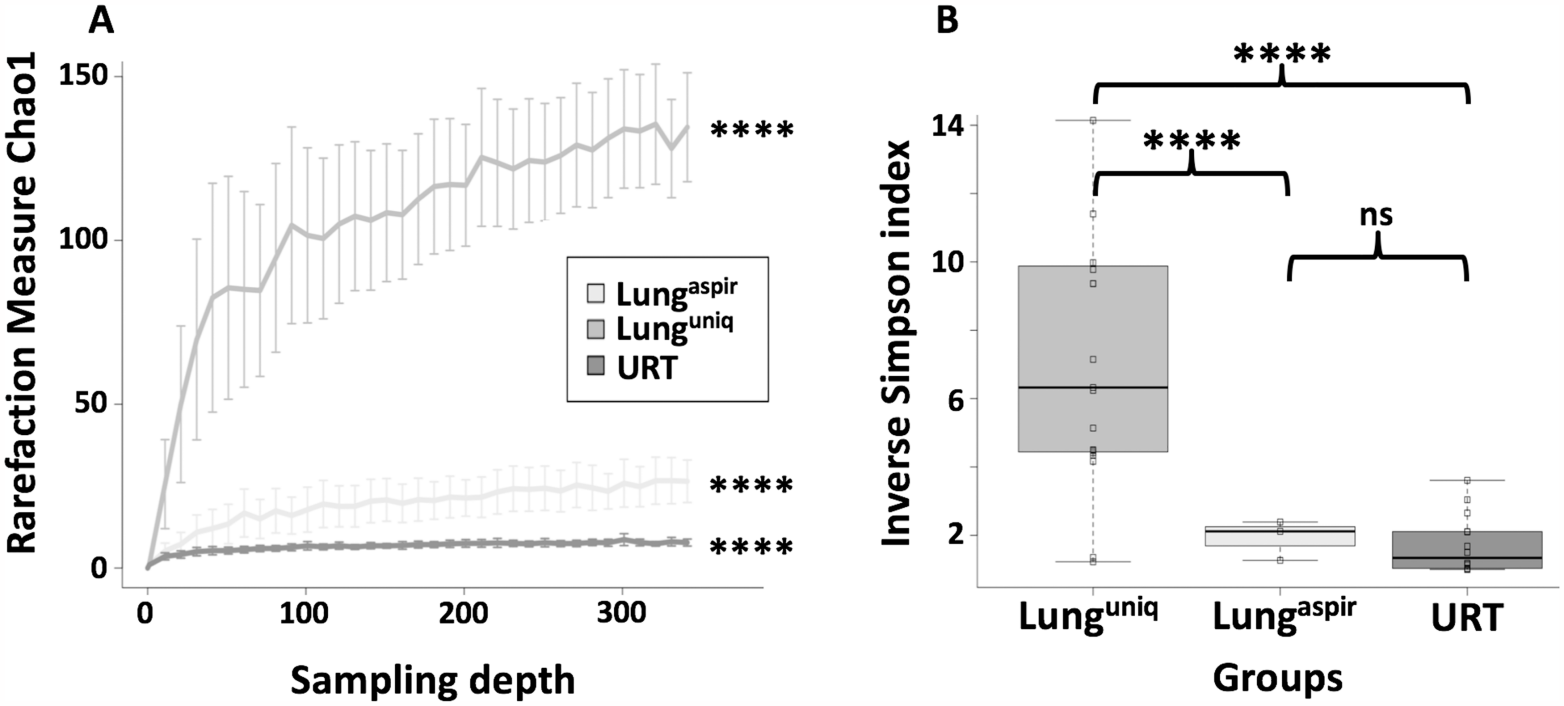
Taxonomic richness of groups was determined at the genus level. A: Each point on the curve represents the mean + SD of 50 Chao1 indices computed for each of 50 random samplings at the indicated sampling depth. B: The Inverse Simpson index was calculated as described in [31]. It measures the probability that two randomly selected genera from different samples will be non-identical. Differences between groups were determined using Student’s t-test. ****; p < 0.0001.

Two indices were used to evaluate the number of different taxa present within and between groups (known as alpha diversity). The number of distinct taxa present in each group was assessed using the *Chao1* measure (Fig 2A) [30]. The *Chao1* index (computed by sub-sampling to a final depth of 340 reads) was highest for the lung^unique^ group, suggesting that it contains more distinct taxa than the URT or lung^aspirate^ groups. The rarefaction curves plateau at about 100 reads, which indicates this sampling depth as sufficient for subsequent group comparison. The *Inverse Simpson* index was used to assess variability within groups, as it combines a richness matrix with a measure of evenness of abundances from different taxa [31]. The lung^unique^ group yielded a significantly higher *Inverse Simpson* index than the URT or lung^aspirate^ groups p < 0.0001 (Fig 2B).

### Analysis of beta-diversity

**Fig 3:**
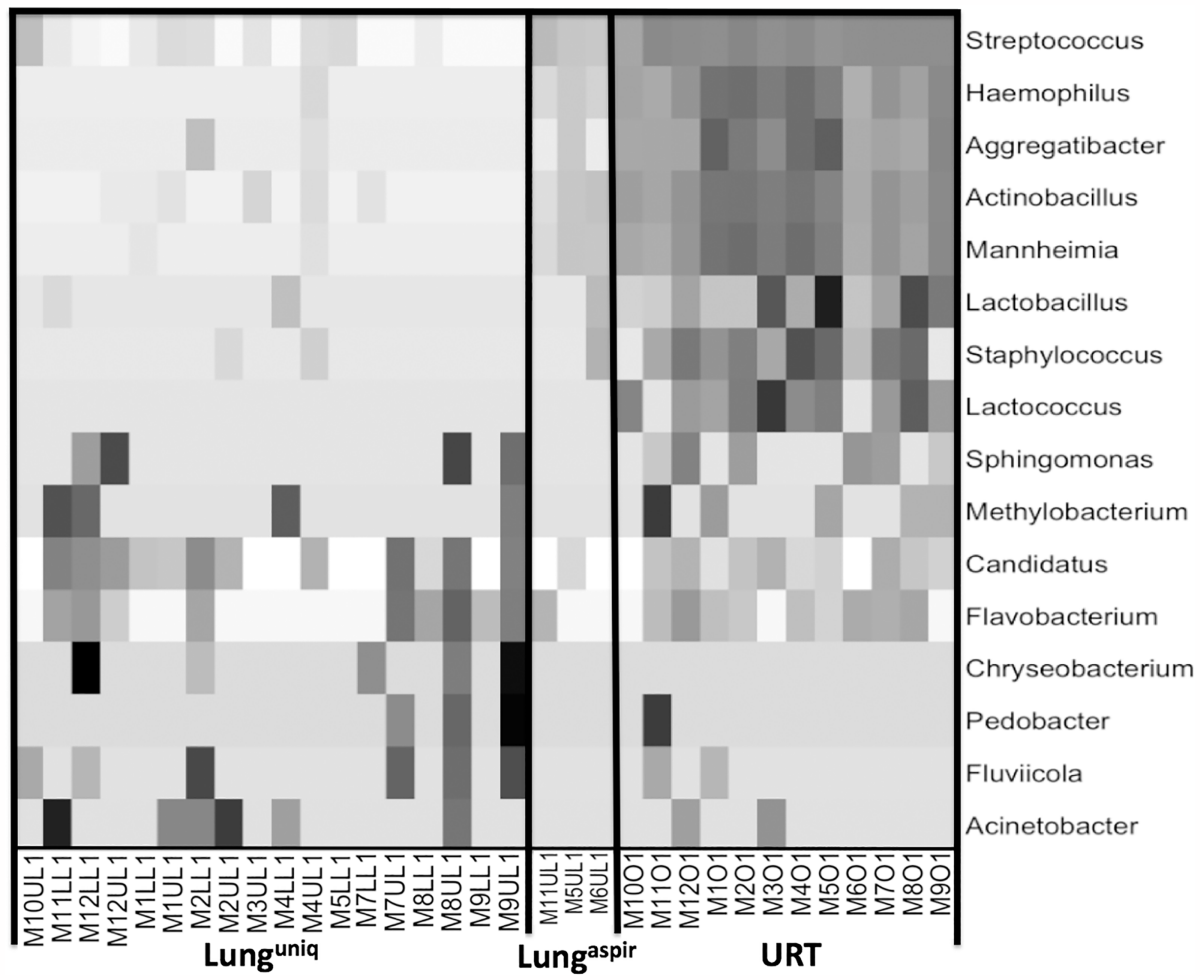
Heatmap. A heatmap was generated using the log_2_ normalized number of sequences for genera with sample abundances >1%. The bacterial genera are shown in the rows and the individual samples are shown in the columns labeled by mouse of origin. Density of gray coloration reflects the relative frequency of each genus to the mean for that genus.

A heatmap was generated to facilitate visual comparison of sample groups. Each group clustered in a pattern consistent with that predicted by the PCOA. The URT was enriched in the genera *Streptococcus*, *Haemophilus*, *Aggregatibacter*, *Actinobacillus*, *Mannheimia*, *Lactobacillus*, *Staphylococcus*, *Lactococcus*, *Sphingomonas* and *Methylobacterium*. The lung^unique^ group was characterized by the presence of *Candidatus*, *Flavobacterium* and *Chryseobacterium* and to a lesser extent *Pedobacter*, *Fluviicola* and *Acinetobacter*. The lung^aspirate^ group contained members of both groups and was enriched in *Streptococcus*, *Haemophilus*, *Aggregatibacter*, *Actinobacillus*, *Mannheimia* (which is characteristic of the URT) as well as with *Candidatus* and *Flavobacterium* (which is primarily found in lung^unique^ samples, Fig 3).

### Distribution of genera among groups

**Fig 4:**
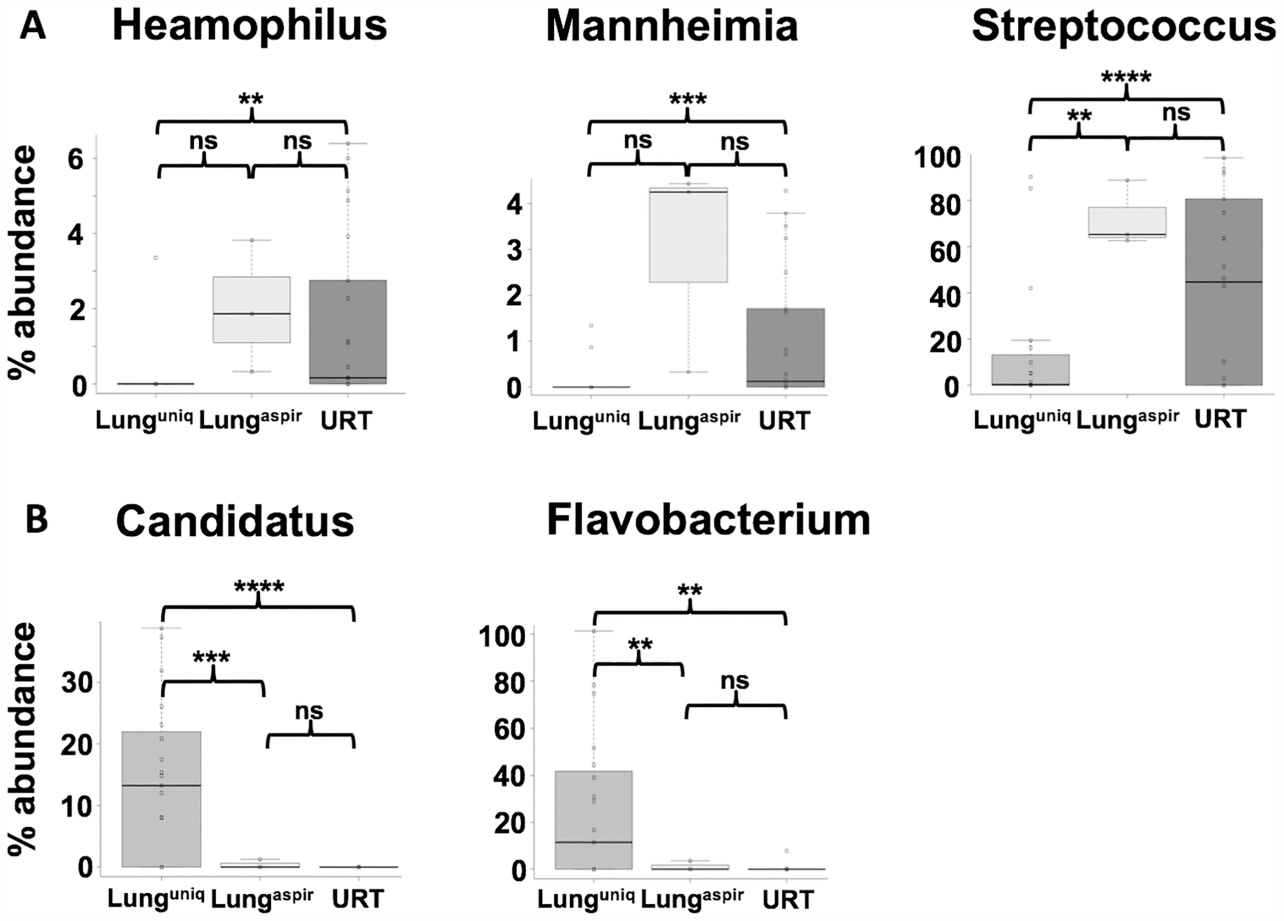
The relative abundance of common URT and lung^unique^ genera. A: Differences between groups were determined by significant ANOVA using data from Fig 3. Genera are shown and characterized by their significantly higher relative abundances in URT samples at an abundance of > 1%. B: The genera are shown as characterized by their significantly higher abundances in lung^unique^ samples with an abundance of > 1%. **; p < 0.01, ***; p < 0.001, ****; p < 0.0001, ns; not significant, Wilcox test.

*Haemophilus*, *Mannheimia* and *Streptococcus* accounted for > 90% of all sequence reads in the URT samples. These genera were also present at higher frequency in lung^aspirate^ but not lung^unique^ samples (from which they were nearly absent, Fig 4A). The opposite pattern was found for *Candidatus* and *Flavobacterium*: these genera were present at significantly higher frequencies in lung^unique^ samples but were largely absent from the URT (Fig 4B).

#### Comparison with environmental samples

To identify the origin of the bacterial DNA present in the lungs and URT, microbial DNA isolated from mouse food, bedding and drinking water was analyzed and the degree of overlap with tissue samples calculated (Table 4). Samples from the lung^unique^ group showed strong overlap (> 50%) with the microbiome present in cage bedding and food samples (dominated by the genera *Acinetobacter* and *Flavobacterium*) and no overlap with drinking water. URT samples showed overlap with the microbiome found in cage bedding (> 50%, dominated by *Streptococcus*), correlated to a lesser extend with rodent chow (38%) but not with the drinking water (Table 4).

**Table 4:** Overlap between sample groups and environmental microbiomes.

Environmental samples	Percent Overlap		
	Lung^unique^	Lung^aspirate^	URT
**Cage Bedding**	52	23	52
**Drinking Water**	3	4	0.8
**Rodent Chow**	60	17	38

Environmental samples were taken from used cage bedding, drinking water and rodent chow (N = 3/group). The percentage overlap between the groups and environmental samples was calculated as described in Methods: the average number of sequences per genus was multiplied by the fraction of samples in which that genus was present. The outcome for all genera was summed and divided by the total number of sequences per group.

## Discussion

The lung is constantly exposed to microbes present in the upper respiratory tract and suspended in the air. Nevertheless, the lung has historically been considered a sterile site because viable organisms could rarely be cultured from healthy pulmonary tissue. That perspective changed with the advent of culture-independent next generation sequencing methods that detected bacterial DNA from a wide variety of genera in the lungs of multiple mammalian species.

Lung tissue is a source of "low bacterial biomass" samples since the overwhelming majority of DNA they contain is of host (including nuclear and mitochondrial genomes) rather than bacterial origin. Microbial DNA contamination of samples/reagents can therefore overwhelm the signal generated by bacteria present in the lungs. In this context, many studies involving pulmonary DNA fail to include sequence data from all relevant controls, confounding interpretation of their results. In this study, lungs were removed aseptically through the thoracic cavity with sterile and UV treated surgical instruments, potential contamination from reagents identified, and then computationally eliminated from the data set to facilitate comparison between the lung and URT of 18 healthy Balb/cJ mice. Results show that a highly conserved microbiome is present in the URT of co-housed mice (overlap > 70%, Table 3). This microbiome was dominated by the genera *Streptococcus*, *Haemophilus*, *Aggregatibacter*, *Actinobacillus*, *Mannheimia*, *Lactobacillus*, *Staphylococcus*, *Lactococcus*, *Sphingomonas* and *Methylobacterium*. In contrast, lung samples from individual mice expressed distinct microbiomes. Iterative comparisons between samples suggested that lung tissues could be categorized into three groups: i) lung^background^; having few or no detectable microbes, ii) lung^aspirate^; expressing a microbiome similar to the URT and iii) lung^unique^; comprising a microbiota distinct from that of the URT.

A third of all lung samples (33%) fell into the lung^background^ group. Identifying members of this group required that sequences introduced by reagent contamination and other sources of artifact were removed from the data set. That procedure also enabled our identification of the weak signal characteristic of the lung^unique^ group (58%). The lung^unique^ pneumotype is dominated by *Candidatus*, *Flavobacterium* and *Chryseobacterium*, all of which are gram negative and either obligate or facultative aerobes. Indeed, whereas aerobes constitute > 60% of the lung^unique^ microbiome they represent less than 1% of the microbiome in the URT and lung^aspirate^ groups (S3 Table). This is consistent with high oxygen levels present in the lungs selecting for the growth of aerobic bacteria.

The lung^unique^ group exhibits greater heterogeneity between mice and overall greater alpha-diversity than the other groups. The lung^unique^ group is qualitatively similar in diversity to the human lung [32;33], but has not been identified as a pneumotype in humans [34]. This may reflect either a real difference between mice and humans or the use of disparate techniques to pool data and detect/remove contaminating sequences.

Whereas 58% of samples were classified as "lung^unique^" the remaining 9% of the lung samples were identified as the "lung^aspirate^" group. While the microbiome of the lung^aspirate^ group resembles that of the URT and is dominated by a few abundant genera (e.g. *Streptococcus*), it also contained bacterial DNA typical of the lung^unique^ group, e.g. *Candidatus* and *Flavobacterium*. The lung^aspirate^ group may provide a potential model of human pulmonary disease as it exhibits the same low level of alpha diversity and is similarly dominated by a few abundant genera.

Previous sequencing studies pooled data from multiple lung samples to generate an "average" microbiome. That statistical approach inadvertently over-emphasized the contribution of URT-derived bacteria (which are present at high frequency in a minority of samples) to yield a microbiome that is non-representative of most pulmonary tissue samples. Thus, the conclusion by some groups that human lung microbiota derives primarily from the upper respiratory tract, in accordance with the “adapted island model of biogeography” [5], must be reconsidered in the light of our finding that the murine lung actually encompasses three distinct pneumotypes. Environmental and anatomical differences between the lung and URT support the expectation that these sites should contain different bacterial communities. Facultative anaerobes such as *Streptococcus* and *Haemophillus* might prosper in the deep gingival areas, dental plaques and gums of the URT but not under the consistently high oxygen tension characteristic of ventilating lung [35]. Moreover, genera such as *Streptococcus* colonize the URT at such high density that they inhibit the growth of other species, a situation absent from low biomass lung tissue [36]. Compared to the URT, nutrient availability in the lungs is very low and the lungs are covered by a mucous layer that contains bacteriostatic surfactant and immune cells designed to prevent microbial colonization [37;38]. These observations are consistent with our finding that the frequency and type of bacteria present in the lung differs from that of the URT.

To clarify the additional sources of lung bacterial DNA, samples from cage bedding, drinking water and rodent chow were evaluated. A majority of the bacterial DNA present in the URT and lung^aspirate^ groups was also present in cage bedding, consistent with the coprophagic behavior of mice [39]. In contrast, the lung^unique^ microbiome most closely resembled and clustered with environmental samples found in rodent chow and cage bedding (Table 4). We postulate that fragments of chow and bedding are inhaled by mice since they eat from the ground and their heads are raised to reach food stored in the cage lid. The influence of other factors on the development and persistence of the pulmonary microbiome, such as gender and housing conditions, was not investigated in this work and could be the target of future investigations [11].

It is important to recognize that viable bacteria may not be the source of most bacterial DNA in the lungs. Pezzulo et al. studied this issue in pig lungs and found that approximately 95% of microbial DNA was DNase I sensitive, indicating that the source of the DNA was not replication competent [40]. Willis and colleagues found that up to 50% of the bacterial DNA from sinus tissue was similarly derived from non-viable sources [41]. As rodent chow and cage bedding is autoclaved prior to use, we believe that the bacterial DNA detected in the lung^unique^ group derives primarily from heat killed microbes. This is consistent with our inability to culture the bacterial genera predicted by genomic studies to be present in the lungs despite repeated attempts using appropriate growth conditions (S4 Table). The traditional view that viable bacteria are rarely present in normal healthy lung is consistent with the lung being actively surveilled by immune cells and the observation that 33% of tissue samples were sterile (the lung^background^ group). Segal et al. reported that the bacterial DNA content of nearly 2/3 of human lung samples could not be distinguished from background/negative control samples [34]. These findings support the conclusion that most of the bacterial DNA isolated from lung tissue derives from extracellular, dead, or phagocytosed/non-viable organisms. Such DNA could nevertheless be biologically relevant as CpG motifs present in bacterial DNA can activate the innate immune system via Toll-like receptor 9 [42;43]. Similarly, macrophages, bronchial and alveolar epithelial cells express pathogen recognition receptors that are stimulated by motifs expressed by even non-viable bacteria [44;45].

This work is the first to compare the microbiomes of the lung and URT in mice. The experimental strategy used minimized sample contamination and other confounding biases that might falsely increase taxonomic overlap between the URT and lungs. Results suggest that co-housed mice share a common oropharyngeal microbiome. In contrast, three distinct pneumotypes were identified in the lungs (two of which have also been described in humans). A third of all lung samples were sterile. Others closely resembled the microbiome of the URT. A third group was identified that contained bacterial DNA similar to that found in rodent chow and cage bedding. Yet detecting microbial DNA does not establish that normal healthy lungs are colonized by bacteria: indeed, appropriate culture techniques generally fail to isolate viable microorganisms from pulmonary tissue. We propose that bacteria reach the lungs by aspiration of oral contents and inspiration of food and bedding particles but are rapidly cleared by protective immune mechanisms.

## Supporting information

S1 Table Summary of studies concerning the murine lung microbiome. Overview of published work concerning the murine microbiome. Note the absence of sequence data involving diluent/reagent controls.
(DOCX)

S2 Table Genera present in negative control samples. Eight reagent and three water control samples were analyzed. The table shows therelative abundance of genera present at a frequency of > 1% in these samples (totalnumber of sequences = 9053).
(DOCX)

S3 Table Properties of bacteria in the lung^unique^, lung^aspirate^ and URT groups. The properties characteristic of each bacterium/genus as reported in the literature were assigned based on their frequency in each group. Data show the mean ± SD. P values were determined using Student’s t-test.
(DOCX)

S4 Table Growth of bacteria under various culture conditions. 180 bacterial cultures were set up from 6 independent lung tissue samples. Growth was found in 5 cultures (**+**: tryptic soy plates in aerobic conditions) and all contained the single bacterial species *Rastonia pickettii* (a known water borne contaminant). Abbreviations: LB; Luria broth
(DOCX)

S1 Fig Clustering of lung samples from different lung sites. PCOA plot showing the clustering of lung samples from two lung sites; upper lung (UL) and lower lung (LL). The centroid of the sites clusters coincide as shown by the concentric 95% confidence ellipse for both groups, respectively.
(TIFF)

S2 Fig Expression of bacterial 16SrDNA. qPCR was used to quantify the amount of bacterial 16SrDNA in samples from the URT (N = 11) and lung (N = 13). **** p < 0.0001, Student's t-test.
(TIFF)

S3 Fig Distribution of UniFrac Distances. A: Distribution of distances to the UniFrac group centroids for members of the URT and three pneumotypes described in the text. URT shows the least intra-group variation while lung^background^ shows the greatest. B: Non-metric multidimensional scaling plot showing the clustering of samples on the basis of the weighted UniFrac distances between them. Group designations are the pneumotypes whose derivation is described in the text. Ellipses represent 1 SD envelopes for the groups.
(TIFF)

S4 Fig Laplace Parameter for Dirichlet Multinomial Mixture model. A: The Laplace goodness of fit parameter for Dirichlet Multinomial Mixture model constructed using from 1 to 7 components with exclusion of lung^background^ group. Lower values indicate a better fit to the data. The optimum model is obtained using two components. B: Heatmap showing counts of the most significant taxa for members of the groups obtained using the optimal Dirichlet Multinomial mixture. The columns represent individual samples and the highlighted columns after each group represent the median bacterial abundance. Group 1 is largely a combination of the pneumotypes lung^aspirate^ and URT. Group 2 is comprised of the members of lung^unique^ group.
(TIFF)
