# Supplementary material for: Three distinct pneumotypes characterize the microbiome of the lung in BALB/cJ mice
Source: PLoS One. 2017 Jul 6;12(7):e0180561. doi: 10.1371/journal.pone.0180561 (PMC5500332; doi:10.1371/journal.pone.0180561)
Supplement: S1 Table — Overview of published work concerning the murine microbiome. Note the absence of sequence data involving diluent/reagent controls. (DOCX) [file pone.0180561.s001.docx]

**S1 Table. Summary of studies concerning the murine lung microbiome.**

| **Author, year, Reference** | **Summary of findings** | **Method** | **Inclusion of negative controls** | **Data availability** |
| --- | --- | --- | --- | --- |
| **Poroyko, 2015, [10]** | LPS induced alteration of the mouse lung microbiome was characterized by fewer *Firmicutes* (*Alicyclobacillaceae*); increased *Proteobacteria*: (*Brucellaceae*, *Xanthomonadaceae*). MALDI TOF: Stenotrophomonas maltophilia, Ochrobactrum antropi. | V3-V4, Illumina MiSeq, MALDI TOF | No | Not available |
| **Barfod, 2015, [9]** | Lung and gut microbiomes differ. Lung microbiome is influenced by nasal antibiotics but not oral probiotics. | Denaturing gradient gel electrophoresis (DGGE) | Mock DNA extraction of saline, results not included in analysis | Not available |
| **Barfod, 2013, [6]** | Lung microbiome phyla: *Proteobacteria*, *Firmicutes*, *Actinobacteria*, *Bacteroidetes*, and *Cyanobacteria;genera: Staphylococcus (8.3%), Massilia (2.6%), Corynebacterium (2.2%), Psedomonas (2.5%), Streptococcus (2.3%), Sphingomonas (1.7%).* | V3-V4 | PCR negative control on agarose gel, no sequencing data | PRJNA231159 |
| **Gollwitzer, 2014, [8]** | Lung microbiome genera: *Staphylococcus* (8.3%), *Massilia* (2.6%), Corynebacterium (2.2%), Pseudomonas (2.5%), Streptococcus (2.3%), Sphingomonas (1.7%). | Phylum specific PCR on extracted DNA | No | Not available |
| **Nguyen, 2016, [7]** | Lung microbiome phyla: *Proteobacteria, Firmicutes, Cyanobacteria, Bacterioidetes, Actinobacteria, P. aeroginosa infection alters pulmonary microbiome.* | V4 | No | European Bioinformatics Institute (EBI) ERP01871; qiita.microbio.me ID 10280 |
| **Yun, 2014, [11]** | Lung microbiome phyla: *Proteobacteria, Firmicutes, Actinobacter, Bacterioides, Cyanobacteria* and *Lactobacilles*; genera: *Lactobacillus, Rastonia, Enterobacteriaceae, Sphingomonas, Pasteurella. Pseudomonas pneumotrophica* and *Acinetobacter muris* cultured from non-SPF and wild mice. | V1-V2, 454 Pyrosequencing | *Rastonia*-specific PCR of the 16SrDNA gene, no sequencing on germ free mice | PRJEB7007 |

Overview of published work concerning the murine microbiome. Note the absence of sequence data involving diluent/reagent controls.
