# Supplementary material for: Three distinct pneumotypes characterize the microbiome of the lung in BALB/cJ mice
Source: PLoS One. 2017 Jul 6;12(7):e0180561. doi: 10.1371/journal.pone.0180561 (PMC5500332; doi:10.1371/journal.pone.0180561)
Supplement: S2 Table — Eight reagent and three water control samples were analyzed. The table shows therelative abundance of genera present at a frequency of > 1% in these samples (totalnumber of sequences = 9053). (DOCX) [file pone.0180561.s002.docx]

**S2 Table. Genera present in negative control samples.**

| **Genus** | **Relative Abundance (%)** |
| --- | --- |
| Pseudomonas | 52.7 |
| Escherichia | 14.6 |
| Sphingobium | 5.7 |
| Aeromonas | 3.3 |
| Shewanella | 2.7 |
| Brevundimonas | 2.6 |
| Citrobacter | 2.5 |
| Agrobacterium | 1.7 |
| Acinetobacter | 1.7 |
| Streptococcus | 1.5 |
| Sphingomonas | 1.4 |
| Delftia | 1.3 |
| Actinobacillus | 1.0 |
| Methylobacterium | 1.0 |

Eight reagent and three water control samples were analyzed. The table shows the

relative abundance of genera present at a frequency of >1% in these samples (total

number of sequences = 9053).
