# Supplementary material for: Three distinct pneumotypes characterize the microbiome of the lung in BALB/cJ mice
Source: PLoS One. 2017 Jul 6;12(7):e0180561. doi: 10.1371/journal.pone.0180561 (PMC5500332; doi:10.1371/journal.pone.0180561)
Supplement: S3 Table — The properties characteristic of each bacterium/genus as reported in the literature were assigned based on their frequency in each group. Data show the mean ± SD. P values were determined using Student’s t-test. (DOCX) [file pone.0180561.s003.docx]

**S3 Table. Properties of bacteria in the lung^unique^, lung^aspirate^ and URT groups.**

| **Property** | **Percent of bacteria/group** | | | |
| --- | --- | --- | --- | --- |
|  | **Lung^unique^** | **Lung^aspirate^** | **URT** | **p value** |
| Aerobe | 61 **±** 40 | 0.6 **±** 0.5 | 0.02 **±** 0.02 | < 0.0001 |
| Facultative aerobe | 33 **±** 38 | 96 **±** 2.7 | 98 **±** 1.5 | < 0.0001 |
| Motile | 51 **±** 37 | 0.6 **±** 0.5 | 0.02 **±** 0.02 | < 0.0001 |
| Gram positive | 25 **±** 34 | 80 **±** 15 | 76 **±** 20 | < 0.0001 |
| Gram negative | 75 **±** 34 | 19 **±** 15 | 24 **±** 20 | < 0.0001 |

The properties characteristic of each bacterium/genus as reported in the literature were assigned based on their frequency in each group. Data show the mean ± SD. P values were determined using Student’s t-test.
