# Supplementary material for: Three distinct pneumotypes characterize the microbiome of the lung in BALB/cJ mice
Source: PLoS One. 2017 Jul 6;12(7):e0180561. doi: 10.1371/journal.pone.0180561 (PMC5500332; doi:10.1371/journal.pone.0180561)
Supplement: S4 Table — 180 bacterial cultures were set up from 6 independent lung tissue samples. Growth was found in 5 cultures (+: tryptic soy plates in aerobic conditions) and all contained the single bacterial species Rastonia pickettii (a known water borne contaminant). Abbreviations: LB; Luria broth (DOCX) [file pone.0180561.s004.docx]

**S4 Table. Growth of bacteria under various culture conditions.**

| **Agar type** | **Incubation conditions** | |
| --- | --- | --- |
|  | **Aerobic** | **Anaerobic** |
| **LB** | - | - |
| **Tryptic Soy** | + | - |
| **5% sheep blood** | - | - |

180 bacterial cultures were set up from 6 independent lung tissue samples. Growth was found in 5 cultures (**+**: tryptic soy plates in aerobic conditions) and all contained the single bacterial species *Rastonia pickettii* (a known water borne contaminant). Abbreviations: LB; Luria broth
