# Supplementary figures and images for: Three distinct pneumotypes characterize the microbiome of the lung in BALB/cJ mice
Source: PLoS One. 2017 Jul 6;12(7):e0180561. doi: 10.1371/journal.pone.0180561 (PMC5500332; doi:10.1371/journal.pone.0180561)

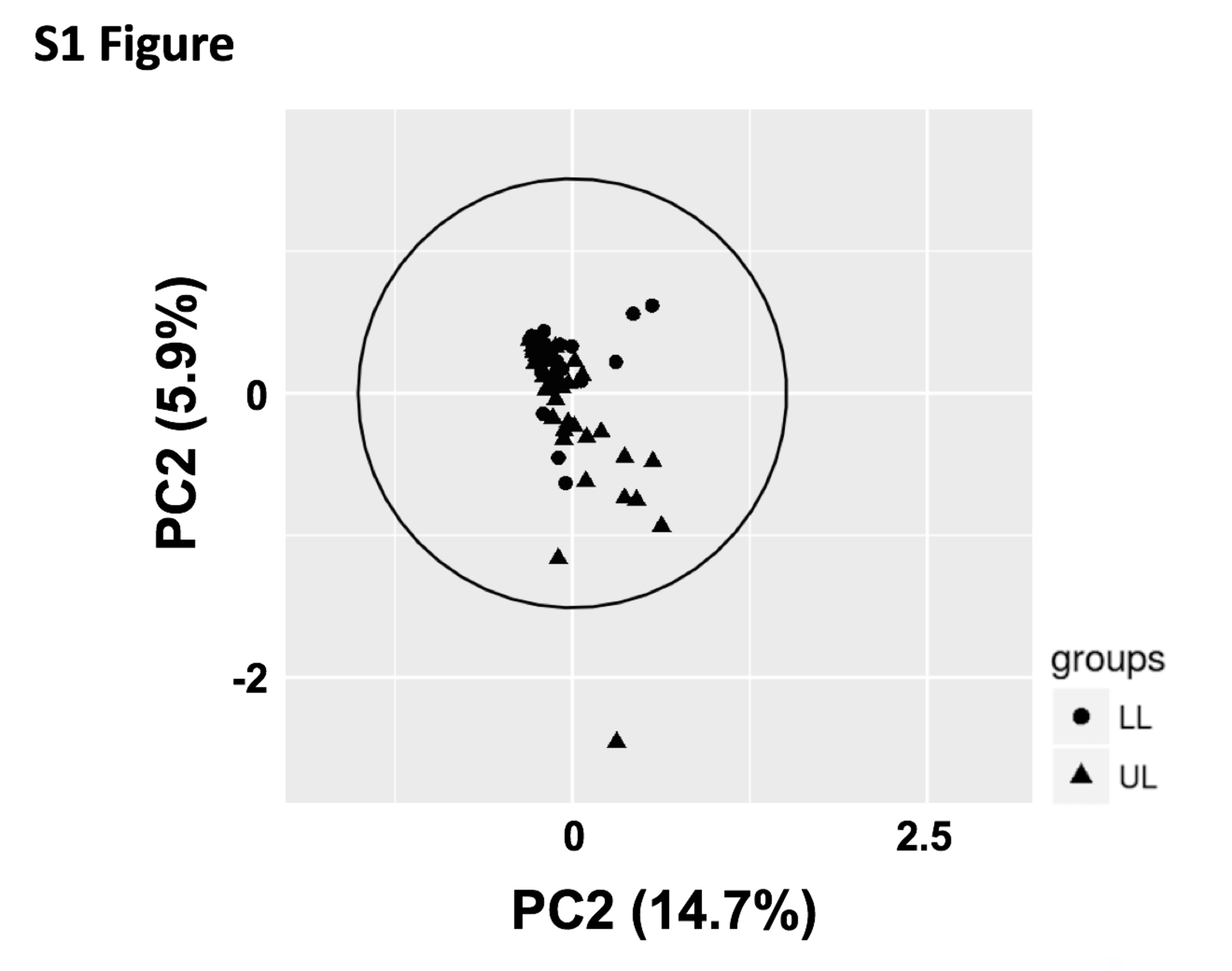

Supplement: S1 Fig — PCOA plot showing the clustering of lung samples from two lung sites; upper lung (UL) and lower lung (LL). The centroid of the sites clusters coincide as shown by the concentric 95% confidence ellipse for both groups, respectively. (TIFF) [file pone.0180561.s005.tiff]

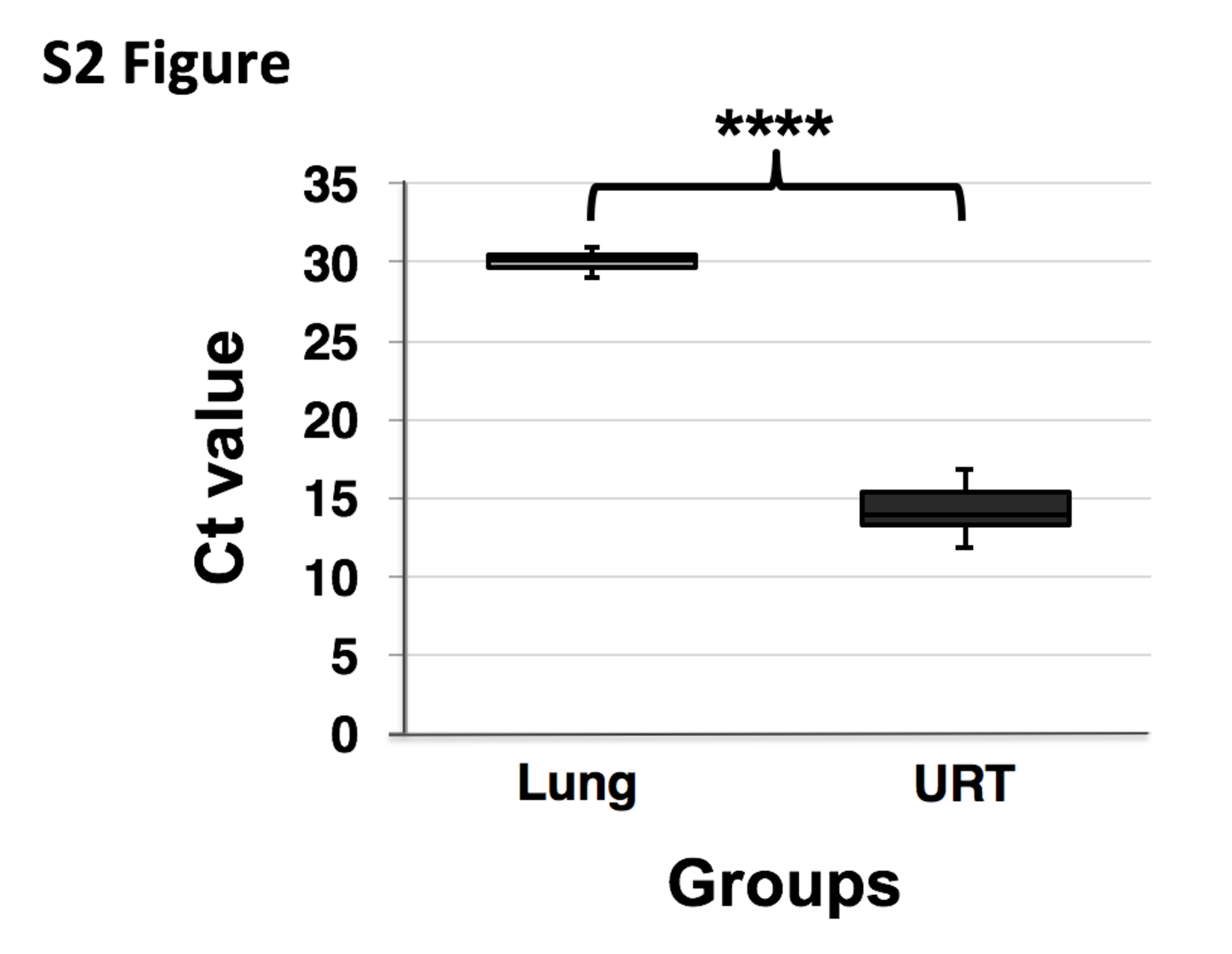

Supplement: S2 Fig — qPCR was used to quantify the amount of bacterial 16SrDNA in samples from the URT (N = 11) and lung (N = 13). **** p < 0.0001, Student's t-test. (TIFF) [file pone.0180561.s006.tiff]

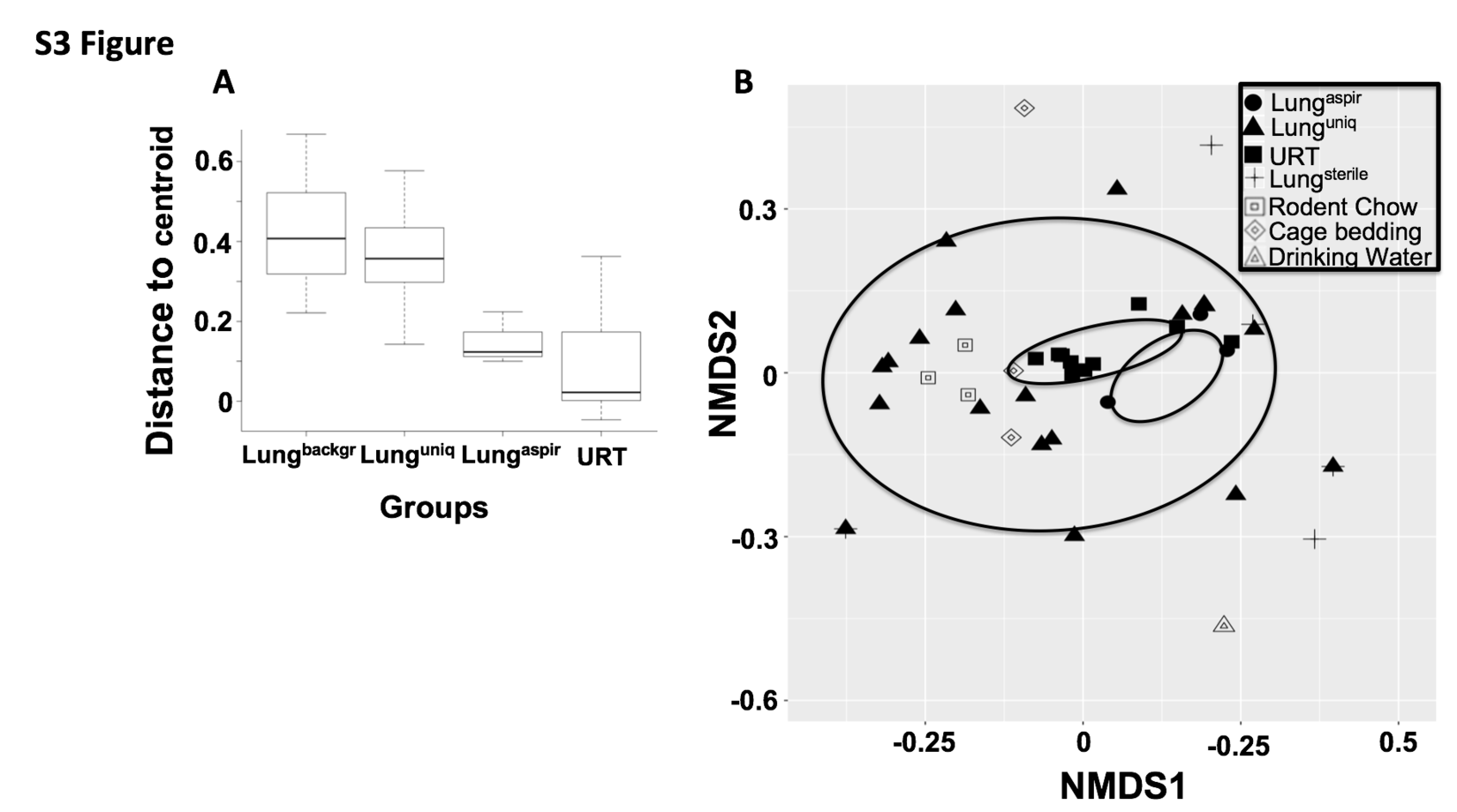

Supplement: S3 Fig — A: Distribution of distances to the UniFrac group centroids for members of the URT and three pneumotypes described in the text. URT shows the least intra-group variation while lungbackground shows the greatest. B: Non-metric multidimensional scaling plot showing the clustering of samples on the basis of the weighted UniFrac distances between them. Group designations are the pneumotypes whose derivation is described in the text. Ellipses represent 1 SD envelopes for the groups. (TIFF) [file pone.0180561.s007.tiff]

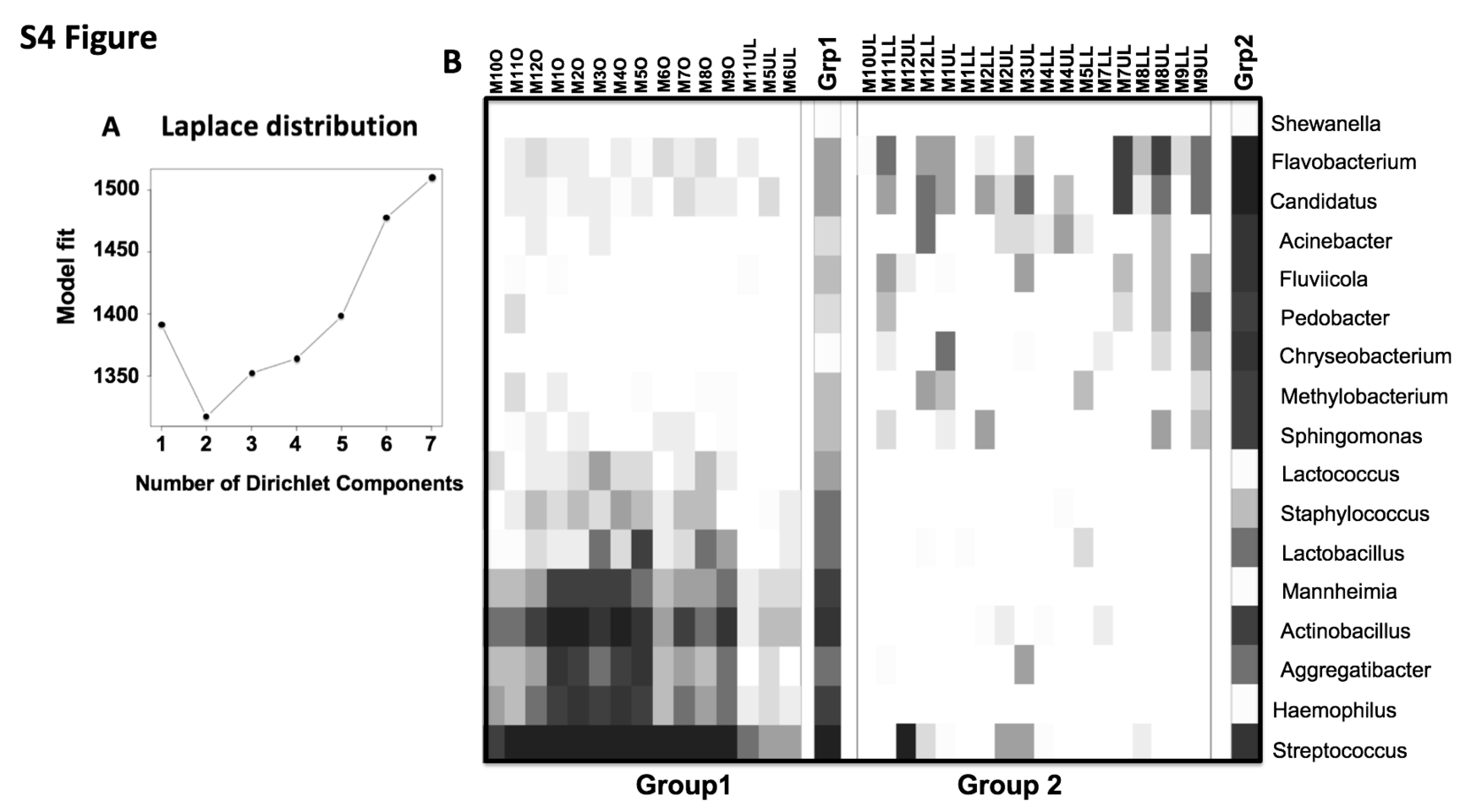

Supplement: S4 Fig — A: The Laplace goodness of fit parameter for Dirichlet Multinomial Mixture model constructed using from 1 to 7 components with exclusion of lungbackground group. Lower values indicate a better fit to the data. The optimum model is obtained using two components. B: Heatmap showing counts of the most significant taxa for members of the groups obtained using the optimal Dirichlet Multinomial mixture. The columns represent individual samples and the highlighted columns after each group represent the median bacterial abundance. Group 1 is largely a combination of the pneumotypes lungaspirate and URT. Group 2 is comprised of the members of lungunique group. (TIFF) [file pone.0180561.s008.tiff]
